# The paradoxical effects of splenectomy on tumor growth

**DOI:** 10.1186/1742-4682-3-23

**Published:** 2006-06-26

**Authors:** Richmond T Prehn

**Affiliations:** 1 Department of Pathology, University of Washington, Seattle, WA, USA

## Abstract

**Background:**

There is a vast and contradictory literature concerning the effect of the spleen and particularly of splenectomy on tumor growth. Sometimes splenectomy seems to inhibit tumor growth, but in other cases it seems, paradoxically, to facilitate both oncogenesis and the growth of established tumors.

**Approach:**

In this essay I have selected from this large literature a few papers that seem particularly instructive, in the hope of extracting some understanding of the rules governing this paradoxical behavior.

**Conclusion:**

In general, whether splenectomy enhances or inhibits tumor growth seems to depend primarily upon the ratio of spleen to tumor. Small proportions of spleen cells usually stimulate tumor growth, in which case splenectomy is inhibitory. Larger proportions of the same cells, especially if they are from immunized animals, usually inhibit tumor growth, in which case splenectomy results in tumor stimulation.

## Spleen cell/tumor cell mixtures

For a general but detailed description of the spleen and its functions see [1].

In one of my own studies, I showed that when a relatively small proportion of spleen cells from specifically immunized donors was admixed with sarcoma cells prior to implantation of the mixture into radiated and thymectomized syngeneic mice, growth of the resulting tumor was relatively stimulated [2]. Larger proportions of the same immune cell population inhibited growth when mixed with the tumor. Non-immune spleen cells or cells that were immune to a different tumor were also stimulatory, but to a significantly much lesser degree. These observations support the conclusion that the immune response to a tumor transplant is biphasic; a quantitatively small spleen-cell response enhances tumor growth, but a larger quantity of the same reactants, relative to the amount of tumor, is inhibitory.

**Figure 1:**
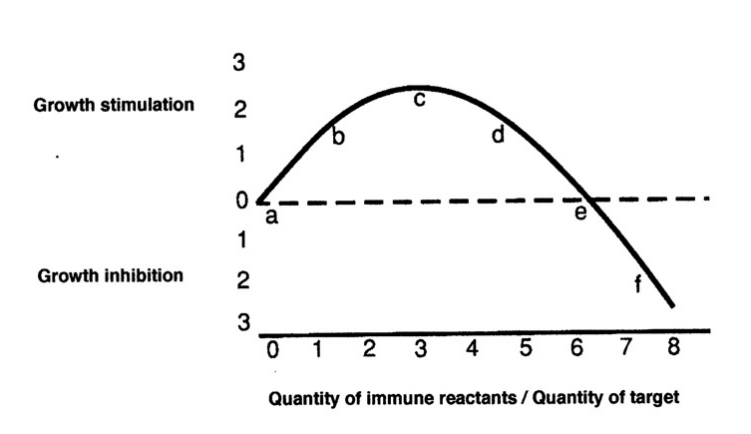
Curve of tumor growth as influenced by proportions of immune reactants.

To reiterate, as illustrated in Figure 1 (which first appeared in [3]), immune spleen cells, and seemingly the very same spleen cells, can be either stimulatory or inhibitory to the growth of an implanted tumor depending upon the quantitative proportions of tumor cells (antigen) to spleen cells. A small ratio of immune spleen cells is stimulatory to tumor growth while a sufficiently large ratio is inhibitory. In this essay, I have defined immunogenicity as the capacity of a prior implant of syngeneic tumor to alter the growth of a subsequent challenge implant of that same tumor.

It is beyond the scope of this essay to discuss in any detail the possible molecular mechanisms by which spleen cells are able to facilitate or inhibit tumor growth, or which among the many cellular species in the spleen may be responsible for these contrasting abilities. However, it may not be amiss to cite a wonderful essay by Harry Rubin, which drew attention to numerous demonstrations of the fact that the degree of cellular aggregation can determine and control the growth and differentiation of cells both *in vitro *and *in vivo *[4]. One may speculate that the varied effects on tumor growth produced by varied proportions of spleen cells might result from an alteration, by splenic elements, of the adhesiveness of the tumor cell membranes, thereby perhaps altering the effective density of a tumor cell population.

## Transplanted tumors

Spleen cells need not be directly mixed with the tumor cells for the spleen to exert a dosage-dependent effect. Implanted tumors are also influenced by the remote spleen as is shown by the effect of prior splenectomy. In one study [5] in which a large dose, 1 × 10^7^, of Meth 1 tumor cells was inoculated, splenectomy reduced tumor growth. In relation to Figure 1, the system apparently fell to the left of "c" on the curve with a larger dose of tumor cells (a lower proportion of spleen cells) and was moved in the direction of "a" by splenectomy. In contrast, when only 5 × 10^5 ^tumor cells were inoculated, splenectomy improved the tumor-takes; with this smaller dose of tumor cells (a higher proportion of spleen cells) the system apparently fell on a position well to the right of "c" and was moved toward "c" by splenectomy. A similar effect of dosage on the activity of the spleen visa-vis a tumor implant was also clearly identified by Nordlund & Gershon [6] and by Chang & Turk [7]. It is apparent that whether splenectomy stimulates or inhibits tumor growth depends upon which side of point "c", in Figure 1, the system lies when the spleen is intact.

## Splenic variation within a single inbred strain

Data suggest a marked variability in susceptibility to chemical sarcogenesis among mice of a single highly inbred strain. The earlier appearance of a methylcholanthrene-induced sarcoma in one animal of a pair marked that mouse as having about a 70% chance of developing the next tumor before its paired mate did so [8]. It was concluded that the increased susceptibility was not caused by the earlier tumor, but that the earlier tumor merely indicated a greater susceptibility to tumor formation that had been present before the first tumor appeared. This conclusion seems justified for five separate reasons. (1) Control mice, which had been exposed to a syngeneic tumor-implant rather than to tumor induction, were not more susceptible to subsequent carcinogenesis [8]. (2) It has been established that immunological cross-reactions among independently induced sarcomas are exceedingly rare [9], so the likelihood that an earlier tumor might influence the appearance of a second independent tumor by immunological means seems remote. (3) Putative reduction of the immunogenicities of the tumors by reducing the carcinogen concentration eliminated the susceptibility differences [10]. (4) Reduction of the immunological capacities of the animals by thymectomy and radiation also eliminated the apparent differences in tumor susceptibility among the mice [10]. (5) Most importantly, as I will soon detail, the variability in susceptibility was transferable from mouse to mouse via spleen cells before any tumor had been induced and before the carcinogen had been administered [11]. Thus, it seems safe to conclude that animals of a single inbred strain do vary markedly, presumably for epigenetic reasons, in their immunological susceptibilities to the induction of immunogenic tumors; a variability that must be attributable, at least in part, to variations in their spleens that predate the administration of carcinogen.

It is well understood that differences among many phenotypic characters depend upon epigenetically determined differences in gene activity rather than actual differences among the genes involved. Epigenetic influences determine whether the same genome will specify a liver as opposed to a nerve cell or a patch of white skin on some C57 Bl mice, but not on others. Thus, it is not surprising to discover that splenic variations, even within an inbred strain, may have a profound effect on chemical oncogenesis. Indeed, while there appear to be many mutations within cancers, many tumors may probably have their genesis in epigenetic aberrations rather than, or as well as, in mutations [12]. The results make it clear that epigenetic factors play a large part in determining the activity of the spleen vis-a-vis chemically-induced cancers; it is epigenetically-induced physiological variation, largely in the spleen, not just chance timing of some transformational event, that determines which animals of an inbred strain get tumors before others.

The experiments showing the transfer of variability from one mouse to another prior to exposure to the carcinogen, and thus prior to the inception of the first tumor, gave an unexpected and seemingly paradoxical result [11]. The experiment was designed for analysis by pairs. Both mice in each initial pair, A and B, were splenectomized and then given a standard subcutaneous dose of methylcholanthrene. The spleen from mouse A was transferred to a third animal, A+, and the spleen from B was transferred to a fourth mouse, B+. A+ and B+ were then also given the carcinogen. In nine out of nine trial pairs (p < 0.001), if a tumor appeared in A before one appeared in B, then a tumor appeared in B+ before one appeared in A+; conversely, if tumor appeared in B before A, the next tumor appeared in A+ before B+. Thus, spleen cells taken from the donor that subsequently (after splenectomy) proved relatively susceptible to oncogenesis conferred a relative resistance to oncogenesis on the recipient; while spleen cells from the other donor, the more resistant of the pair, imparted relative susceptibility on the recipient.

A logical explanation for this seemingly paradoxical result may reside in the fact that in each transfer the whole minced spleen of a single donor was transferred intraperitoneally to a single radiated, thymectomized recipient. Thus, a very significant proportion of the animal's entire lymphoid population was transplanted. If the donor spleen was of a type to confer relatively high susceptibility to oncogenesis on a secondary host, it seems reasonable that the donor of that spleen might be left with a relative paucity of tumor-facilitating capacity. Conversely, a donor that had originally been relatively resistant to oncogenesis probably became relatively susceptible by virtue of splenectomy; but the spleen transferred the donor's original relative resistance to the recipient.

## Other effects on oncogenesis

A number of other studies also show that splenectomy often has profound effects on carcinogenesis. Female rats were splenectomized and then exposed to 9,10-dimethyl-1,2-benzanthracene to induce mammary tumors. Splenectomy decreased the rate of appearance of these immunogenic tumors [13,14]. Other work suggests that chemical oncogenesis produces specific tolerance to, or relative stimulation of, the induced tumor and its antigens [15,16]. Thus, it seems that oncogenesis, at least in systems in which immunogenic tumors are produced (most chemical carcinogen systems), is often subject to inhibition by splenectomy. Prior to splenectomy, these systems probably lie near "b" or "c" on the Figure 1 curve.

By analogy with the already-discussed experiment in which spleen cells were mixed with tumor cells in varying proportions, one might predict that, in systems in which tumors of low immunogenicity are produced, oncogenesis might result in growth-inhibitory rather than stimulatory splenic activity, and that splenectomy would facilitate rather than inhibit tumor growth in such cases. However, it should be realized that there may be two types of non-immunogenic tumors. Looking at Figure 1, it is apparent that there are two places on the curve where a tumor eliciting that particular ratio of reaction would be considered non-immunogenic: at "a" and at "e". If a tumor induces an immune reaction that puts it near "e", any reduction in the quantity of immune reactants, as by splenectomy, would be expected to enhance tumor growth. However, if the immune response places the tumor anywhere between "a" and "c", any such reduction in the proportion of immune reactants would be expected to inhibit tumor growth and/or incidence. I have recently proposed that an immune reaction may be necessary *in vivo *for oncogenesis to occur [17]; if this were really so, it is possible that no tumors could fall directly on point "a".

The preceding analysis appears to be largely consistent with observation. Squartini [18] showed that when tumors appear to be relatively non-immunogenic, as do viral mammary tumors in the mouse, and the effective ratio of immunogen to spleen cells is decreased by splenectomy, tumorigenesis is facilitated. The facilitation of tumor growth suggests that prior to splenectomy this system fell near "e" on the Fig. 1 curve, and the apparent lack of immunogenicity of the tumors was due to a balance between facilitating and inhibiting immune reactions.

In the human, splenectomy for trauma has little if any effect on the subsequent occurrence of cancers [19,20]. If some human tumors are immunogenic while others are much less so, splenectomy will sometimes cause facilitation and sometimes inhibition of tumor development and thus have little net effect. It is also possible that many human tumors might actually fall at or near "a" on the curve. Perhaps a more likely explanation for the absence of any effect of splenectomy on the incidence of tumors in humans is that if there were too long an interval between splenectomy and tumor development, compensatory mechanisms might negate much of the effect of the splenectomy [21].

## Alimentary tract

A paper by Hull et al. [22], which might seem inconsistent with my thesis, examined the effect of splenectomy upon the appearance of 1,2 dimethylhydrazine-induced intestinal tumors in the mouse. Contrary to expectation, splenectomy appeared to enhance the appearance of carcinomas, although it did not increase the incidence of benign lesions. No direct measure of the immunogenicity of the tumors appears to be available, but one usually expects chemically-induced tumors to be immunogenic and to fall between "b" and "c" on the biphasic curve. Two very different explanations seem possible: perhaps the tumors were indeed so immunogenic that they fell to the right of "c"; and/or the tumors were intrinsically immunogenic but the immunity was blocked.

The latter explanation could be considered because of the possible induction of oral tolerance. Orally-fed cancer tissue induces a non-cross-reactive attenuation of cellular anti-tumor host responses [23]. Oral tolerance may be prevented by prior splenectomy [24]. I suggest the following speculative scenario: the intestinal tumors in the Hull experiment [22], especially the more malignant, may have induced oral tolerance. In the animal of origin, they might thus have fallen near "e" on the biphasic curve and appeared to be non-immunogenic; splenectomy would have moved the system toward "c" and therefore resulted in relative facilitation of the tumors.

A further possible problem is presented by a report that implants of two different carcinogen-induced mouse colon cancers were also enhanced rather than inhibited by splenectomy [25]. Oral tolerance cannot be a factor in tumor implants, as opposed to tumor induction. Although the immunogenicities of the carcinomas are again not known, they were probably highly immunogenic and one of them induced splenomegaly. Probably they were so immunogenic that they fell far to the right of "c", perhaps near "f" on the curve in Figure 1, and splenectomy then moved them in the direction of "c".

## Splenectomy as therapy

The possibility that splenectomy is an effective therapy for established cancers has attracted much interest and introduces yet another variable; the timing of splenectomy. Favorable reports from Japan suggesting that splenectomy is beneficial in the course of surgery for stomach cancer may be marginally correct, but subsequent studies have not been encouraging [26,27].

However, splenectomy markedly delayed the course of B16 melanoma growth [28], and Stolfi et al. [29] reported that, in a murine spontaneous mammary cancer system, splenectomy combined with enucleative tumor surgery reproducibly increased the cure rates in comparison to enucleative surgery alone. This mammary tumor system yields tumors with little or no detectable immunogenicity by the classical test of transplantation into putatively immunized mice [30]. In the tumor system employed by Stolfi et al., allowing the tumor to grow large before enucleation and splenectomy might have allowed the spleen to be exposed to sufficient antigen to shift the system from near "e" (non-immunogenic) to "c" or beyond. Splenectomy under these circumstances might then be expected to produce the relative inhibition of tumor growth that was actually observed [29]. This very positive result in what is essentially a non-immunogenic tumor system suggests that therapeutic splenectomy may merit further investigation.

## Miscellaneous effects

Another variable that probably affects the action of the spleen significantly is the age of the organ. The young spleen seems more likely than the spleen from an older animal to exert a facilitating effect upon tumor growth [31]. The very fact that tumors in older animals tend to grow more slowly may reflect the decline with age of the tumor-stimulating capacity of the spleen [32,33].

A number of reports have suggested that perioperative allogeneic transfusion may worsen the prognosis in gastric cancer. Weitz et al. [34] reported that this worsening was mediated by the spleen in a mouse model and was prevented by splenectomy. Splenectomy had no harmful effect in the absence of a blood transfusion. In a somewhat analogous mouse experiment, I found that a large allogeneic blood transfusion profoundly stimulated the growth of, in this case, a transplantable allogeneic tumor. The stimulation was abolished by prior splenectomy [35]. It will be remembered that non-specifically immune or even non-immune spleen cells facilitate tumor growth to some extent when mixed in small proportions with implanted tumor cells [2], so it is perhaps not surprising that allogeneic transfusions might enhance the spleen's facilitation of tumor growth. What role oral tolerance may play in the gastric carcinoma system is uncertain [23].

It has been reported that splenectomy has a differential effect on primary versus metastatic lesions [36] or upon less malignant versus more highly malignant tumors [37]. In the course of liver carcinogenesis, it has been noted that later, more mature hyperplastic nodules grow to form metastasizing hepatocarcinomas if injected into the spleen, but do not grow if injected into numerous other sites such as under the kidney capsule [38]. It is interesting in this connection that Hammond (see [17]) reported that a higher immune capacity in the host promoted tumor progression.

## Conclusion

The effects of the spleen on tumor growth are exceedingly complex. Nevertheless, allowing for a few possibly discordant notes, it appears that most effects can generally be explained on the basis of the quantitative ratio of immune system/antigen: a higher ratio (less tumor and/or antigen) favors inhibition of growth, but a lower ratio (less immune reactants) stimulates the tumor. The facilitating effect of relatively small quantities of immune spleen cells seems to be a positive stimulation of tumor growth and not a mere blocking of tumor inhibition. It is possible that many tumors, considered nonimmunogenic on the basis of classical transplantation tests, may actually elicit an immune response that is balanced between the inhibitory and the tumor-stimulatory properties primarily by the spleen, i.e. they may be at "e" on the biphasic curve. Highly immunogenic tumors would lie at a distance from "a" as well as from "e".

Probably any observation can be accommodated in terms of the biphasic curve in Figure 1, but finding a place for an observation on the curve does not necessarily mean that the interpretation is correct. However, the curve does provide a rational way of thinking about some complex interactions and suggests the need to titrate the immune reaction against tumor size and antigenicity whenever possible.

The promising results of therapeutic splenectomy in several systems suggest that further studies of this phenomenon are desirable, albeit perhaps not with tumors of the alimentary tract.

## References

[B1] Mebius RE, Kraal G (2005). Structure and function of the spleen. Nature Rev Immunol.

[B2] Prehn RT (1972). The immune reaction as a stimulator of tumor growth. Science.

[B3] Prehn RT (1994). Stimulatory effects of immune reactions upon the growth of untransplanted tumors. Cancer Res.

[B4] Rubin H (2006). What keeps cells in tissues behaving normally in the face of myriad mutations?. BioEssays.

[B5] Kumashiro R, Shiraishi M, Sugimachi K, Hiramoto Y, Tamada R, Okamura T, Masuda H, Inokuchi K, Nomoto K (1984). Bidirectional effects of splenectomy on the growth of syngeneic tumor in mice. Jpn J Surg.

[B6] Nordlund JJ, Gershon RK (1975). Splenic regulation of the clinical appearance of small tumors. J Immunol.

[B7] Chang RW, Turk JL (1977). Increased resistance in splenectomized mice to a methylcholanthrene-induced tumour. Br J Cancer.

[B8] Prehn RT (1975). Non-genetic variability in susceptibility to oncogenesis. Science.

[B9] Basombrio MA (1970). Search for common antigenicities among twenty-five sarcomas induced by methylcholanthrene. Cancer Res.

[B10] Prehn RT (1979). Immunological basis for differences insusceptibility to hydrocarbon oncogenesis among mice of a single genotype. Int J Cancer.

[B11] Prehn RT, Karcher CA (1983). Splenic variations affecting sarcomagenesis among mice of an inbred strain. Int J Cancer.

[B12] Prehn RT (1994). Cancers beget mutations versus mutations beget cancers. Cancer Res.

[B13] Kossoy G, Ben-Hur H, Lifschitz O, Zusman I (2002). Mammary tumors in splenectomized rats. Oncol Rep.

[B14] Ben-Hur H, Kossoy G, Lifschitz O, Zusman I (2002). Splenectomy, chemically-induced mammary tumors and parathymic lymph nodes in rats: experimental and morphological studies. In Vivo.

[B15] Stjernswärd J (1968). Immune status of the primary host toward its own methylcholanthrene-induced sarcomas. J Natl Cancer Inst.

[B16] Basombrio MA, Prehn RT (1972). Immune status of autochthonous and adoptively protected mice toward spontaneous and chemically induced tumors. Cancer Res.

[B17] Prehn RT (2006). An adaptive immune reaction may be necessary for cancer development. Theor Biol Med Model.

[B18] Squartini F (1971). Mouse mammary tumorigenesis by mammary tumor virus in the absence of thymus, spleen or both organs. Israel J Med Sci.

[B19] Mellemkjaer L, Olsen JH, Linet MS, Gridley G (1995). Cancer risk after splenectomy. Cancer.

[B20] Linet MS, Nyren O, Gridley G, Mellemkjaer L, McLaughlin JK, Olsen JH, Adami HO, Fraumeni JF (1996). Risk of cancer following splenectomy. Int J Cancer.

[B21] Yamagishi H, Pellis NR, Kahan BD (1980). Effect of splenectomy upon tumor growth: characterization of splenic tumor-enhancing cells in vivo. Surgery.

[B22] Hull CC, Galloway P, Gordon N, Gerson SL, Hawkins N, Stellato TA (1988). Splenectomy and the induction of murine colon cancer. Arch Surg.

[B23] Larkin J, Tangney M, Collins C, Casey G, O'Brien MG, Soden D, O'Sullivan GC (2005). Oral immune tolerance mediated by suppressor T cells may be responsible for the poorer prognosis of foregut cancers. Med Hypotheses.

[B24] Suh ED, Vistica BP, Chan CC, Raber JM, Gery I, Nussenblatt RB (1993). Splenectomy abrogates the induction of oral tolerance in experimental autoimmune uveoretinitis. Curr Eye Res.

[B25] Sato N, Michaelides MC, Wallack MK (1983). Effect of splenectomy on the growth of murine colon tumors. J Surg Oncol.

[B26] Ertuck S, Ersan Y, Cicek Y, Dogusoy G, Senocak M (2003). Effect of simultaneous splenectomy on the survival of patients undergoing curative gastrectomy for proximal gastric carcinoma. Surg Today.

[B27] Fatouros M, Roukos DH, Lorenz M, Arampatzis I, Hottentrott C, Encke A, Kappas AM (2005). Impact of spleen preservation in patients with gastric cancer. Anti Cancer Res.

[B28] Fotiadis C, Zografos G, Aronis K, Troupis TG, Gorgoulis VG, Sechas MN, Skalkeas G (1999). The effect of various types of splenectomy on the development of B-16 melanoma in mice. Int J Investig.

[B29] Stolfi RL, Stolfi LM, Fugmann RA, Martin DS (1977). Augmented therapeutic results elicited by splenectomy in combined modality treatment of spontaneous murine breast cancer. Cancer Immunol Immunother.

[B30] Prehn RT, Main JM (1957). Immunity to methylcholanthrene-induced sarcomas. J Natl Cancer Inst.

[B31] Itzhaki O, Skutelsky E, Kaptzan T, Sinai J, Michowitz M, Huszar M, Leibovici J (2003). Ageing-apoptosis relation in murine spleen. Mech Ageing Dev.

[B32] Donin N, Sinai J, Michowitz M, Hiss J, Nordenberg J, Leibovici J (1995). Role of immune response as determinant of tumor progression in function of host age in the B16 melanoma. Mech Ageing Dev.

[B33] Donin N, Sinai J, Staroselsky A, Mahlin T, Nordenberg J, Leibovici J (1997). Comparison of growth rate of two B16 melanomas differing in metastatic potential in young versus middle-aged mice. Cancer Invest.

[B34] Weitz J, D'Angelica M, Gonen M, Klimstra D, Coit DG, Brennan MF, Karpeh MS (2003). Interaction of splenectomy and perioperative blood transfusions on prognosis of patients with proximal gastric and gastroesophageal junction cancer. J Clin Oncol.

[B35] Prehn RT (1959). The immunity-inhibiting role of the spleen and the effect of dosage and route of antigen administration in a homograft reaction. Colloque International sur les Problèmes biologiques des Greffes.

[B36] Michowitz M, Donin N, Sinai J, Leibovici J (1995). Comparison of splenectomy effects as an indication for host response to growth of primary and metastatic tumour cells in two murine tumour systems. Int J Exp Pathol.

[B37] Leibovici J, Michowitz M, Argaman H (1990). Change in the role of the spleen from protective to harmful following tumor progression in AKR lymphoma. Invasion Metastasis.

[B38] Tatematsu M, Lee G, Hayes MA, Farber E (1987). Progression in hepatocarcinogenesis: Differences in growth and behavior of transplants of early and late hepatocyte nodules in the rat spleen. Cancer Res.

